# FAM83A signaling induces epithelial-mesenchymal transition by the PI3K/AKT/Snail pathway in NSCLC

**DOI:** 10.18632/aging.102163

**Published:** 2019-08-24

**Authors:** Fengrui Zhou, Jianxiong Geng, Shanqi Xu, Qingwei Meng, Kexin Chen, Fang Liu, Fang Yang, Bo Pan, Yan Yu

**Affiliations:** 1 Department of Medical Oncology, Harbin Medical University Cancer Hospital, Harbin, Heilongjiang 150081, China; 2 Department of Pathology, Harbin Medical University Cancer Hospital, Harbin, Heilongjiang 150081, China

**Keywords:** non-small cell lung cancer, FAM83A, EMT

## Abstract

Family with sequence similarity 83, member A (FAM83A), as a potential tumor promoter, was reported to contribute to the progression of several malignant tumors. However, the significance of FAM83A in invasion and metastasis of non-small cell lung cancer (NSCLC) remains largely unknown. In this study, we found that FAM83A expression was significantly increased in NSCLC tissues. High expression of FAM83A was positively associated with tumor metastasis and poor survival of NSCLC patients. Functional experiments revealed that FAM83A knockdown could suppress NSCLC cell migration and invasion both *in vivo* and *in vitro*. While opposite results were observed in FAM83A-transfected cells. Mechanically, we found that FAM83A promoted NSCLC cell migration and invasion by inducing epithelial-mesenchymal transition (EMT) via PI3K/ATK/Snail signaling. Rescue experiment demonstrated that inhibition of either AKT or Snail could partially counteract the promoting effect of FAM83A overexpression in NSCLC metastasis. Taken together, our findings are the first time to demonstrate that increased expression of FAM83A in NSCLC was correlated with EMT and tumor metastasis, which may provide a novel therapeutic target in NSCLC treatment.

## INTRODUCTION

Lung cancer remains the most prevalent cause of global cancer-related mortality, leading to over a million deaths each year [1]. Despite some dramatically advances in multimodal therapeutic strategies over the last decade [2], the prognosis is still unfavorable [3]. More than 79% of lung cancer patients develop metastasis, and the 5-year survival rate of these patients is less than 5% [4]. Metastasis accounts for poor prognosis and high mortality rates in non-small lung cancer (NSCLC) [5]. There is an urgent need for better understanding the molecular mechanisms underlying NSCLC metastasis and leveraging potential therapeutic targets for NSCLC prevention and treatment.

Epithelial-mesenchymal transition (EMT) is a critical process for epithelial cells to acquire a mesenchymal phenotype that is associated with tumor metastasis [6–9]. A hallmark of EMT is the functional loss of E-cadherin and store of Vimentin and N-cadherin (mesenchymal markers) [10–12]. It is often regulated by several signal pathways and transcription factors such as Snail and Twist [13–16]. A major signaling pathway that facilitates EMT is via the PI3K/AKT [17–19]. Numerous studies have focused on complex regulatory networks that modulate EMT to explain and discover new mechanisms involved in cancer progression and metastasis.

Family with sequence similarity 83, member A (FAM83A), located on chromosome 8q24, is the smallest member of the FAM83 family (FAM83A to H). It was first implicated as a potential cancer biomarker in 2005 [20]. The conserved DUF1669 domain at its N-terminus was thought to be involved in tumor progression [21–22]. Prior studies had indicated that the elevated expression of FAM83A occurs in a substantial fraction of cervical, testicular, breast, pancreatic, ovarian and bladder cancers [23–28]. Lee et al. [29] reported that FAM83A could trigger the PI3K/AKT pathway to facilitate metastasis of breast cancer by interacting with PI3Kp85. Moreover, Liu et al. [30] showed that FAM83A mRNA was overexpressed in the circulating tumor cells (CTCs) of lung adenocarcinoma (LUAD) patients. Shi et al. [31] suggested that the long noncoding antisense RNA FAM83A-AS1 could promote lung cancer cell progression along with increased FAM83A expression *in vitro*. However, the specific oncogenic abilities and the molecular mechanism of the FAM83A gene in EMT and the metastasis of NSCLC remain largely elusive.

In the present report, we observed that FAM83A was dramatically overexpressed in NSCLC clinical tissues and was associated with metastatic clinicopathologic features and a poor prognosis in NSCLC patients. Most strikingly, we discovered that FAM83A promoted NSCLC cell migration and invasion by inducing EMT via PI3K/ATK/Snail signaling. Inhibition of either AKT or Snail could partially abolish the promoting effect of FAM83A overexpression in NSCLC metastasis. Taken together, our studies provide the first evidence that FAM83A promotes the metastasis of NSCLC and may represent a potential clinical target for cancer therapy.

## RESULTS

### The TCGA indicated that FAM83A was overexpression in NSCLC and might correlate with more aggressive characteristics and a worse prognosis

**Figure 1:**
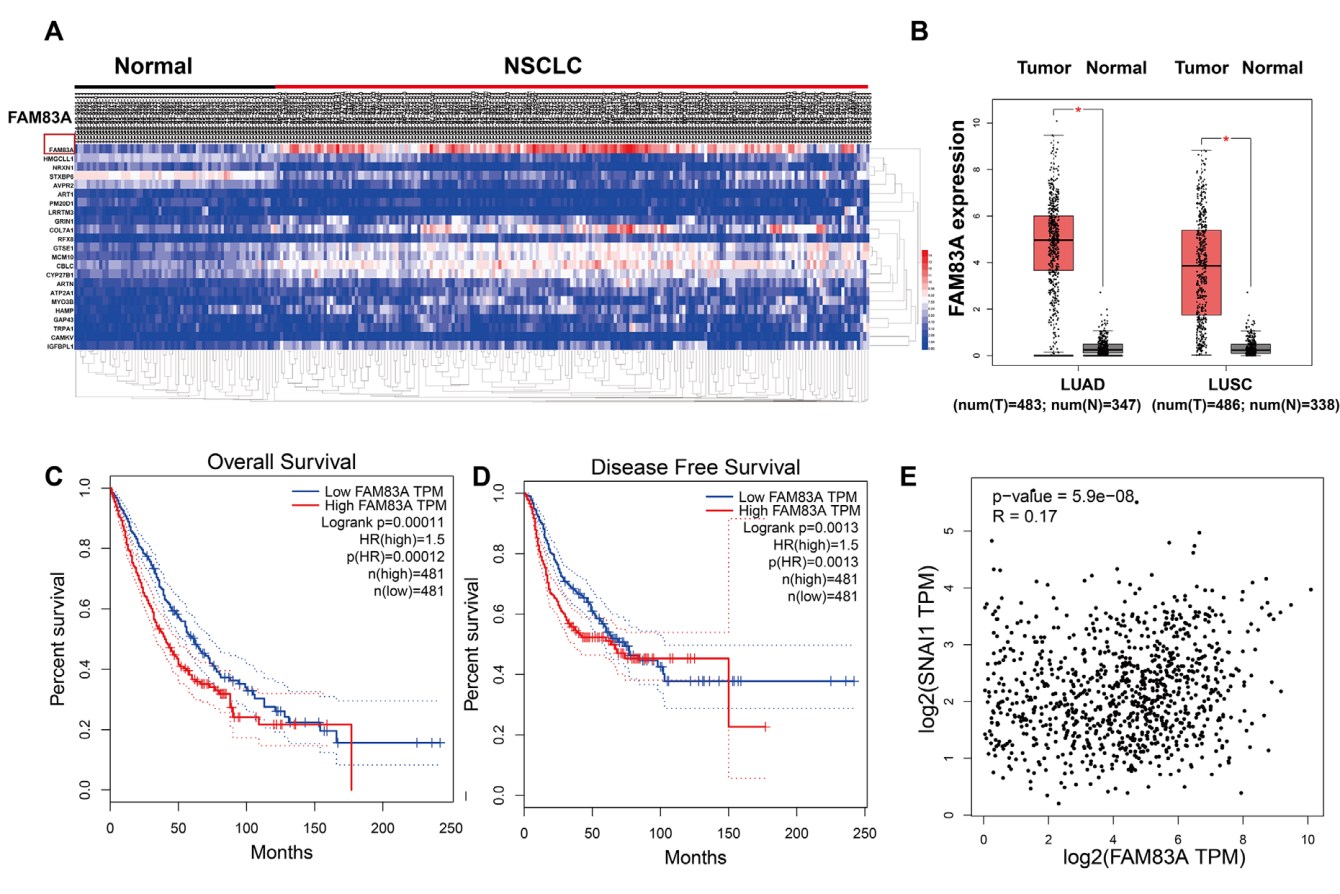
**FAM83A was overexpression in NSCLC tissues and correlated with more aggressive clinical characteristics in the TCGA dataset.** (**A**) Heatmaps showing the clustering patterns of differentially expressed FAM83A between normal and lung cancer specimens. (**B**) Box plot analysis of the FAM83A mRNA levels in lung adenocarcinoma (LUAD) and human lung squamous cell carcinoma (LUSC) tissue samples. (**C**–**D**) Kaplan-Meier plots of overall-survival (**C**) and disease-free survival (**D**) in NSCLC patients with high and low levels of FAM83A. The dotted line indicates the 95% confidence interval. (**E**) Pearson’s test showed that FAM83A had weak positive correlation with Snail (r = 0.17, P<0.001) in NSCLC tissues.

The Cluster Analysis of the Cancer Genome Atlas (https://cancergenome.nih.gov) dataset (Figure 1A) showed a significant presence of FAM83A in lung cancer tissues compared with normal tissues. Similarly, as shown in Figure 1B, the mRNA level of FAM83A was overexpression in NSCLC but rarely detectable in normal lung tissues. Moreover, Kaplan-Meier analysis of NSCLC tissues from TCGA database suggested that patients with higher FAM83A expression had worse overall survival and disease-free survival (P<0.001, P=0.0013; Figure 1C–1D), suggesting that FAM83A may promote progression of NSCLC. Moreover, a correlation analysis in the TCGA dataset showed that FAM83A had a weak positive correlation with the EMT-related transcription factor Snail (Figure 1E) (r = 0.17, P<0.001) in NSCLC tissues.

### Immunohistochemical staining confirmed that high expression of FAM83A in NSCLC was associated with metastatic clinicopathologic features and a poor prognosis

**Figure 2:**
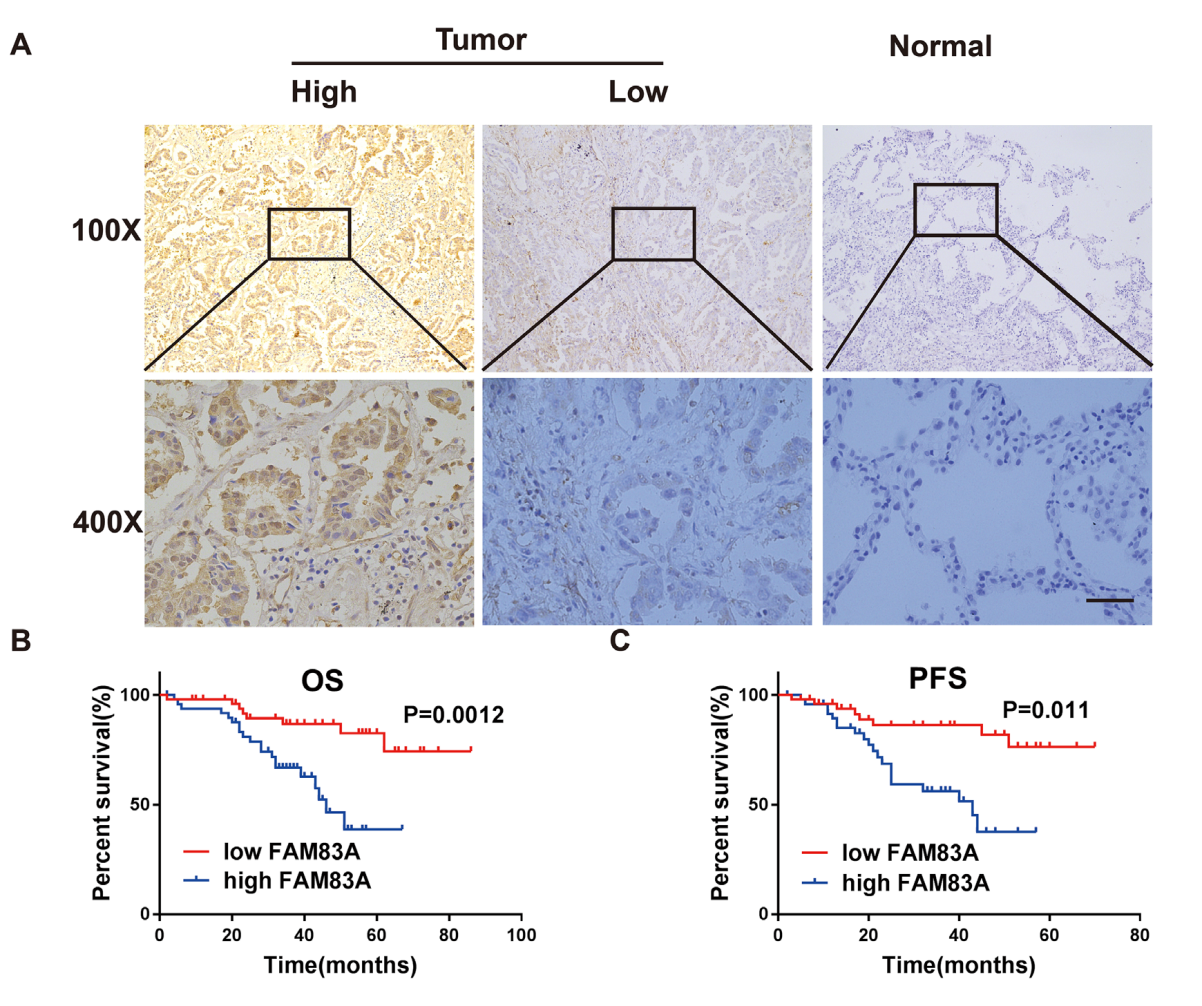
**FAM83A was highly expressed in NSCLC tissues and correlated with worse survival*.*** (**A**) Immunohistochemical staining showed that FAM83A was highly expressed in NSCLC tumors (n = 49/101) compared with normal lung tissues (n = 2/50, *P* < 0.05). (**B**–**C**) Kaplan-Meier plots of overall-survival (**B**) and progression-free survival (**C**) in NSCLC patients with high and low levels of FAM83A. Scale bar, 100 μm.

Consistent with the bioinformatics analysis, Immunohistochemistry results confirmed that FAM83A was markedly upregulated in NSCLC tissues (n=49/101) but barely detectable in normal lung tissues (n =2/50; Figure 2A). Moreover, the expression levels of FAM83A were associated with lymph node involvement (p=0.03), tumor distant metastasis (p=0.012) and clinical stage (p=0.004) (Table 1). There was no significant difference between FAM83A expression levels and tumor size, gender or smoking status.

**Table 1:** Correlations between FAM83A expression and clinical/pathological features in NSCLC.

**Characteristics**		**N**	**FAM83A**		**X^2^**	**P value**
			**High**	**Low**		
Gender	Female	47	24	23		
	Male	54	25	29	0.229	0.633
Age	≤65	81	38	43		
	>65	20	11	9	0.420	0.517
Smoking status	Never	52	25	27		
	ever	49	24	25	0.008	0.928
Lymph node status	Positive	70	39	31		
	Negative	31	10	21	4.733	0.030*
Primary Tumor size	T1	51	25	26	1.453	0.693
	T2	36	16	20		
	T3-T4	14	8	6		
Tumor stage	I	29	9	20		
	II	40	17	23		
	III	32	23	9	11.118	0.004*
Distant metastasis	Positive	55	33	22	6.377	0.012*
	Negative	46	16	30		

Distant metastasis refers to the distant metastasis after TNM staging; *Signifcant correlation.

Kaplan-Meier analysis and the log-rank test revealed that patients with high FAM83A expression had worse overall survival (OS, p=0.0012; Figure 2B) and progression-free survival (PFS, p=0.011; Figure 2C). Moreover, uni- and multi-variate analysis indicated that FAM83A expression level, distant metastasis and smoking were each determined to be independent prognostic indicators of overall survival in NSCLC patients (Table 2). Collectively, these results indicate that FAM83A is markedly correlated with a poor prognosis in NSCLC patients and probably boosts metastasis and progression of lung cancer.

**Table 2:** Univariate and multivariate analysis for OS in patients with NSCLC.

**Characteristics**	**Univariate analysis**			**Multivariate analysis**		
	**HR**	**95%CI**	**P value**	**HR**	**95%CI**	**P value**
Gender	0.901	0.426–1.909	0.786	0.492	0.208–1.164	0.107
Age	1.077	0.409–2.838	0.880	1.121	0.368–3.418	0.840
Smoking status	0.535	0.246–1.166	0.116	0. 276	0.111–0.685	0.006 *
Lymph node status	0.352	0.133–0.931	0.035*	0.895	0.174–4.594	0.894
Primary Tumor size	0.910	0.540–1.536	0.725	0.591	0.335–1.043	0.070
Tumor stage	2.174	1.293–3.658	0.003*	1.483	0.539–4.079	0.446
Distant metastasis	0.295	0.124–0.703	0.006*	0.306	0.108–0.867	0.026 *
FAM83A	0.270	0.116–0.630	0.002*	0.378	0.148–0.971	0.043 *

Distant metastasis refers to the distant metastasis after TNM staging; HR: hazard ratio; CI: confidence interval; *P < 0.05

### FAM83A promoted lung cancer cell metastasis *in vitro*

**Figure 3:**
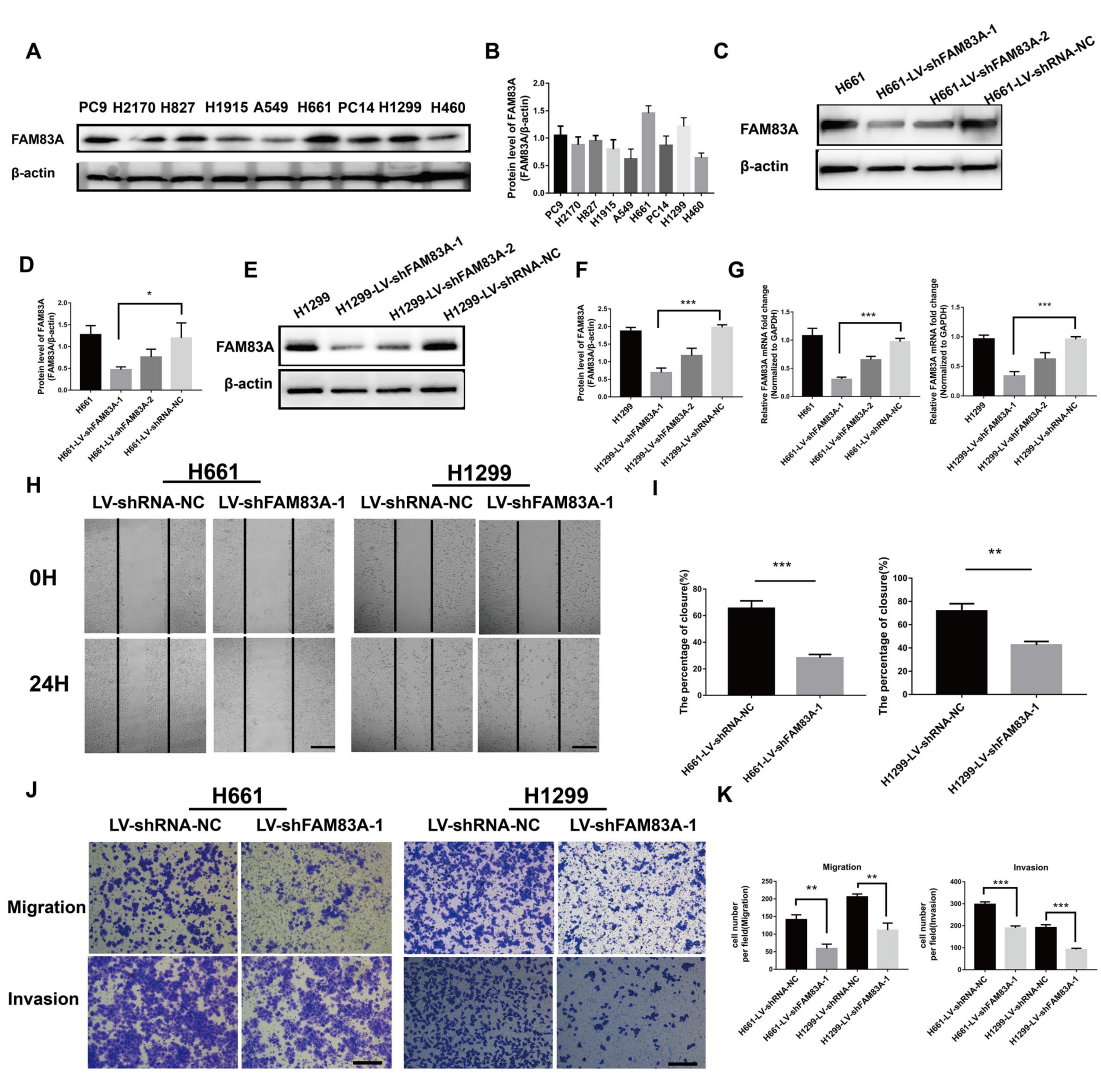
**Suppression of FAM83A inhibited NSCLC cell migration and invasion capacity *in vitro*.** (**A**) FAM83A protein level was assessed in NSCLC cell lines using Western blot analysis. β-actin was used as a loading control. (**B**) The intensity of protein levels was quantified using Image Lab 5.2.1 software and normalized to β-actin. (**C**–**G**) H661 and H1299 cells were transduced with FAM83A lentivirus (LV-shFAM83A-1/2) and a scrambled shRNA lentivirus (LV-shRNA-NC). The suppression of FAM83A in H661 and H1299 cells was confirmed at both the protein (**C**–**F**) and RNA (**G**) levels. (**H**–**I**) H661-shRNA-NC, H661-shFAM83A-1, H1299-shRNA-NC, and H1299-shFAM83A-1 cells were subjected to a wound-healing assay. Images were taken at 0 h and 24 h. (**J**–**K**) Transwell assays assessed tumor cell migration and invasion capacity in FAM83A-knockdown H661 and H1299 cell lines. Scale bar, 200 μm. Error bars: mean ± SD (n=3). *p<0.05, **p<0.01, and ***p<0.001 were considered to indicate a statistically significant difference.

**Figure 4:**
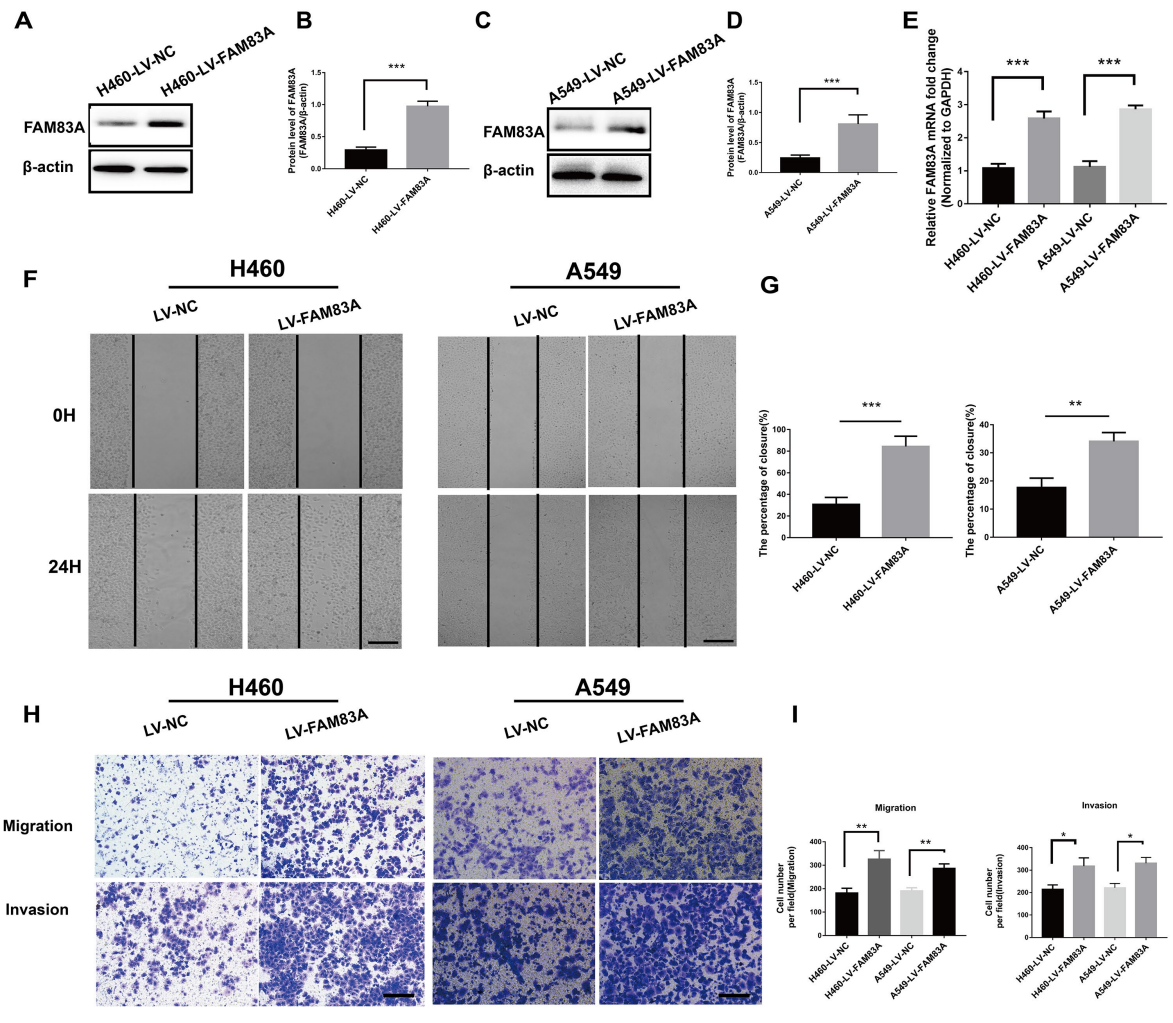
**Overexpression of FAM83A facilitated NSCLC cell migration and invasion capacity *in vitro*.** H460 and A549 cells were transduced with a FAM83A lentivirus (LV-FAM83A) or a control lentivirus (LV-NC). (**A**–**E**) Overexpression of FAM83A in H460 and A549 cells was confirmed at both the protein (**A**–**D**) and RNA (**E**) levels. (**F**–**G**) H460 and A549 cells transduced with the FAM83A lentivirus were subjected to a wound-healing assay. Images were taken at 0 h and 24 h. (**H**–**I**) Transwell assays assessed tumor cell migration and invasion capacity. Scale bar, 200 μm. Error bars: mean ± SD (n=3). *p<0.05, **p<0.01, and ***p<0.001 were considered to indicate a statistically significant difference.

To further investigate the effect of FAM83A on cell migration and invasion *in vitro*, H661 and H1299 cells were chosen to perform stable FAM83A knockdown (Figure 3A–3G, Supplementary Figure 1), while H460 and A549 cells perform stable FAM83A overexpression (Figure 4A–4E). The suppression and overexpression of FAM83A in these cells were confirmed at both the protein and RNA levels. FAM83A knockdown in H661 and H1299 cells significantly impaired cell lateral migration ability, as revealed in a wound-healing assay (Figure 3H–3I). Additionally, Transwell assays demonstrated that FAM83A-knocked down cells migrated and invaded less efficiently than did control cells (Figure 3J–3K). Moreover, exogenous FAM83A introduction into H460 and A549 cells conferred significantly enhanced metastatic potential, as indicated by the enhanced migratory and invasive abilities of these cells in both wound-healing and Transwell assays (Figure 4F–4I). Together, these data suggest that FAM83A promotes metastasis in lung cancer *in vitro*.

### FAM83A induced EMT in NSCLC cells

**Figure 5:**
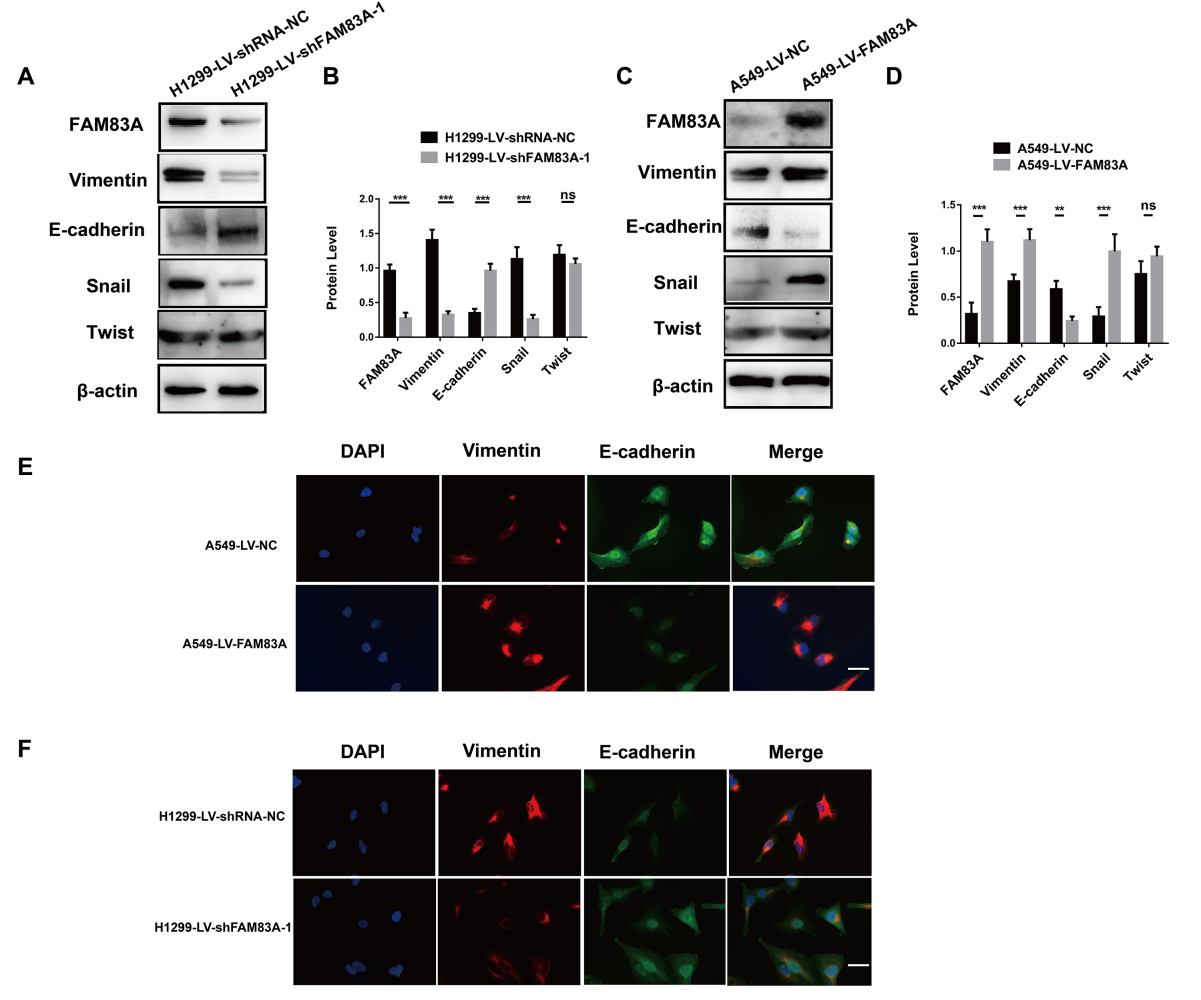
**FAM83A facilitated NSCLC cell EMT progression.** (**A**–**D**) Protein levels of FAM83A, Vimentin, E-cadherin, Snail, and Twist were detected by Western blot in stable H1299-LV-shRNA-NC, H1299-LV-shFAM83A-1, A549-LV-NC and A549-LV-FAM83A cells. β-actin was used as a loading control. (**E**–**F**) Immunofluorescence of Vimentin and E-cadherin in A549 and H1299 cells after manipulation of FAM83A expression. The nucleus was counterstained with DAPI. The fourth panel of each shows the merged image of the previous panels. Scale bar, 100 μm. Error bars: mean ± SD (n=3). NS, no significant, *p<0.05, **p<0.01, and ***p<0.001 were considered to indicate a statistically significant difference.

It has been clearly established that EMT is a vital step in metastasis that could up-regulate the invasive activity of tumor cells [32, 33]. Therefore, we investigated whether FAM83A promoted the protein level of EMT-related markers. As shown in Figure 5A-5B, after depletion of FAM83A, Vimentin and Snail were decreased, while the epithelial marker E-cadherin was increased. Exogenous FAM83A exerted the opposite effects (Figure 5C–5D). However, the down- or upregulation of FAM83A did not influence Twist protein expression (Figure 5A–5D). In addition, immunofluorescence staining showed that overexpression of FAM83A decreased E-cadherin but increased Vimentin in A549 cells (Figure 5E), whereas FAM83A knockdown reverted the EMT phenotype in H1299 cells (Figure 5F). These results indicate that FAM83A induces EMT in NSCLC cells.

### FAM83A induced EMT and metastasis in NSCLC cells by activating the PI3K/AKT/Snail pathway

**Figure 6:**
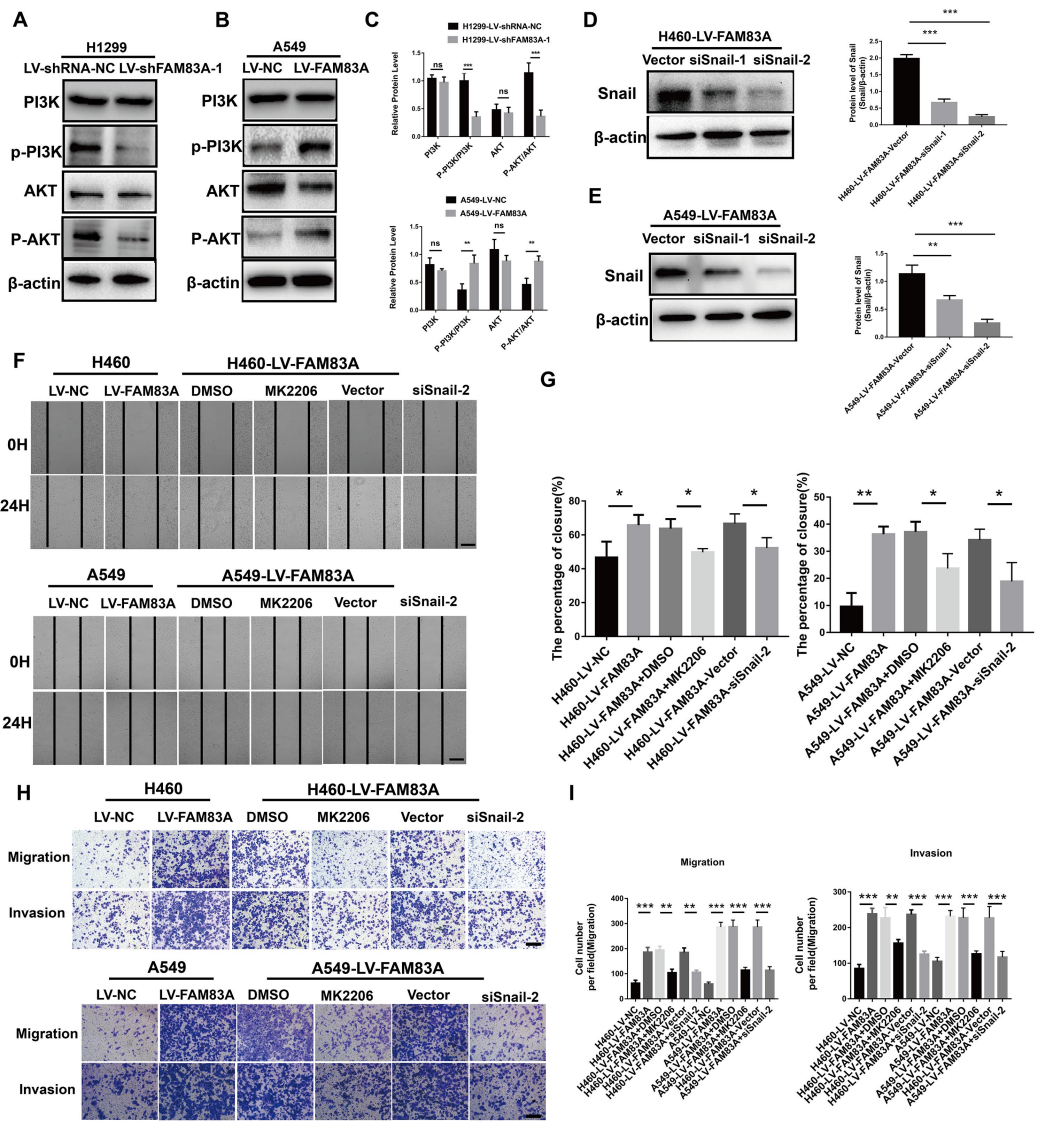
**FAM83A regulated NSCLC cell EMT progression through the PI3K/AKT/Snail pathway.** (**A**–**C**) Protein levels of PI3K, P-PI3K p85 subunit (p-Y458), AKT, and P-AKT (p-S473) were detected by Western blot in stable H1299-LV-shRNA-NC, H1299-LV-shFAM83A-1, A549-LV-NC and A549-LV-FAM83A cells. β-actin was used as a loading control for PI3K and AKT. (**D**–**E**) H460 and A549 with stable FAM83A overexpression cells were transduced with Snail siRNAs (siSnail-1/2) and a scrambled siRNA (Vector). The suppression of Snail in those cells was confirmed by Western blot. (**F**–**G**) FAM83A-transfected H460 and A549 cells (with or without inhibition of MK2206 or Snail) were subjected to a wound-healing assay. Images were taken at 0 h and 24 h. (**H**–**I**) Transwell assay was used to evaluate the effects of MK2206 and Snail knockdown on cell migration and invasion. Scale bar, 200 μm. Error bars: mean ± SD (n=3). *p<0.05, **p<0.01, and ***p<0.001 were considered to indicate a statistically significant difference.

**Figure 7:**
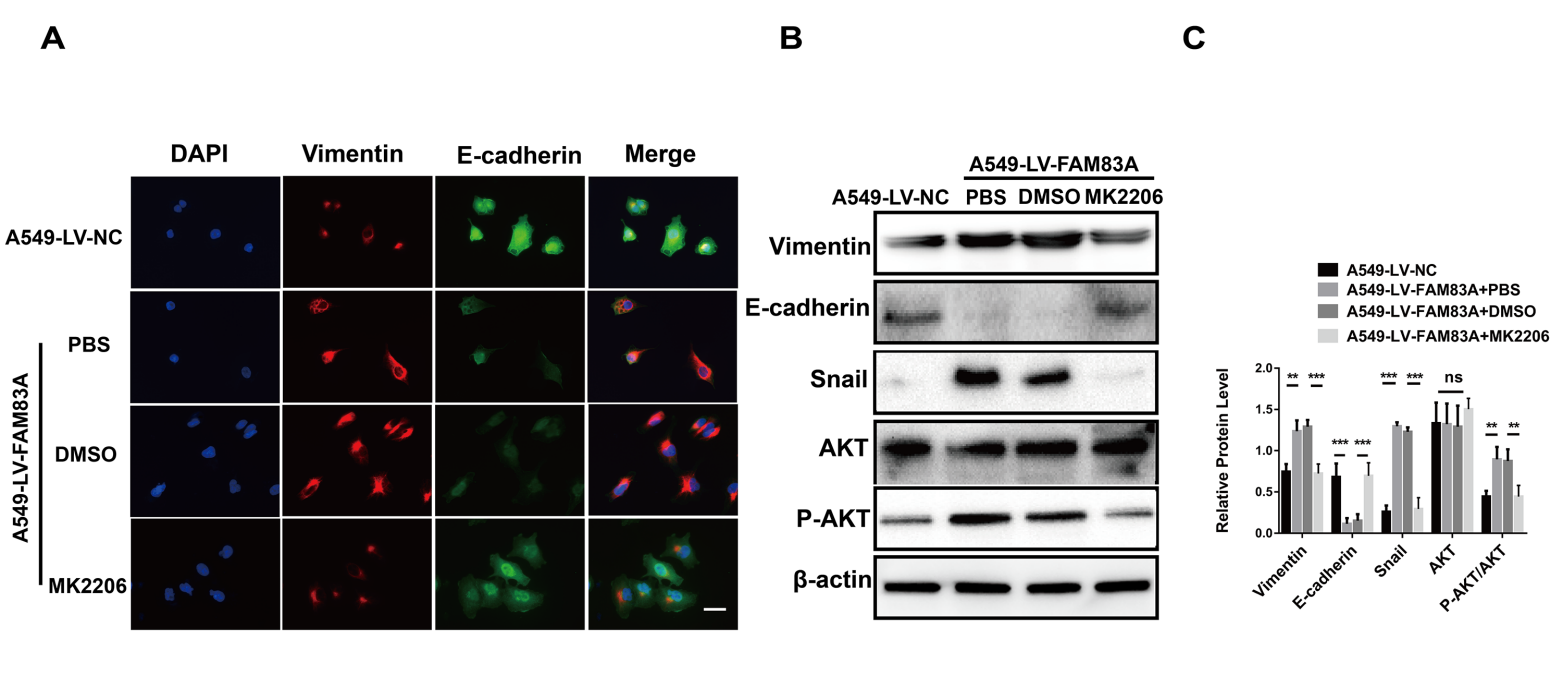
**Inhibition of AKT impaired the EMT phenotype owing to FAM83A overexpression.** (**A**) Immunofluorescence of Vimentin and E-cadherin in FAM83A-transfected A549 cells with or without MK2206. The nucleus was counterstained with DAPI. The fourth panel of each shows the merged image of the previous panels. (B–C) Protein levels of Vimentin, E-cadherin, Snail, AKT and P-AKT (p-S473) were detected by Western blot in stable A549-LV-NC and A549-LV-FAM83A cells (with or without MK2206). Scale bar, 100 μm. Error bars: mean ± SD (n=3). NS, no significant, *p<0.05, **p<0.01, and ***p<0.001 were considered to indicate a statistically significant difference.

Considering the previously established induction role of PI3K/AKT in cancer EMT, we hypothesized that FAM83A promoted metastasis via PI3K/AKT pathway. Therefore, we investigated whether the depletion of FAM83A inhibited the constitutive phosphorylation of PI3K and AKT. As shown in Figure 6A and 6C, p-PI3K and p-AKT were significantly inactivated in shFAM83A-treated H1299 cells. In contrast, FAM83A overexpression in A549 cells reversed the attenuation of p-PI3K and p-AKT (Figure 6B, 6C). Furthermore, in order to verify the role of AKT and important transcription factor Snail in FAM83A-mediated metastasis, we used an AKT inhibitor MK2206 or knocked down the Snail on FAM83A-transfected cells (Figure 6D, 6E). Cells treated with MK2206 or with Snail knocked down showed attenuated migration and invasion owing to FAM83A overexpression in chamber assays (Figure 6F–6I). Moreover, the activity of EMT, which was increased by FAM83A, was receded upon MK2206 treatment (Figure 7A–7C). In order to further confirm FAM83A promotes metastasis through the AKT pathway, the FAM83A was overexpressed after the inhibition of AKT (1μM MK2206). We found that overexpression of FAM83A can restore the metastasis ability of cells, as well as P-AKT expression (Supplementary Figure 2A–2D). Taken together, we proposed a new FAM83A pathway (PI3K/AKT/Snail pathway) promoting lung cancer metastasis and EMT.

### FAM83A promoted lung cancer metastasis and EMT *in vivo*

**Figure 8:**
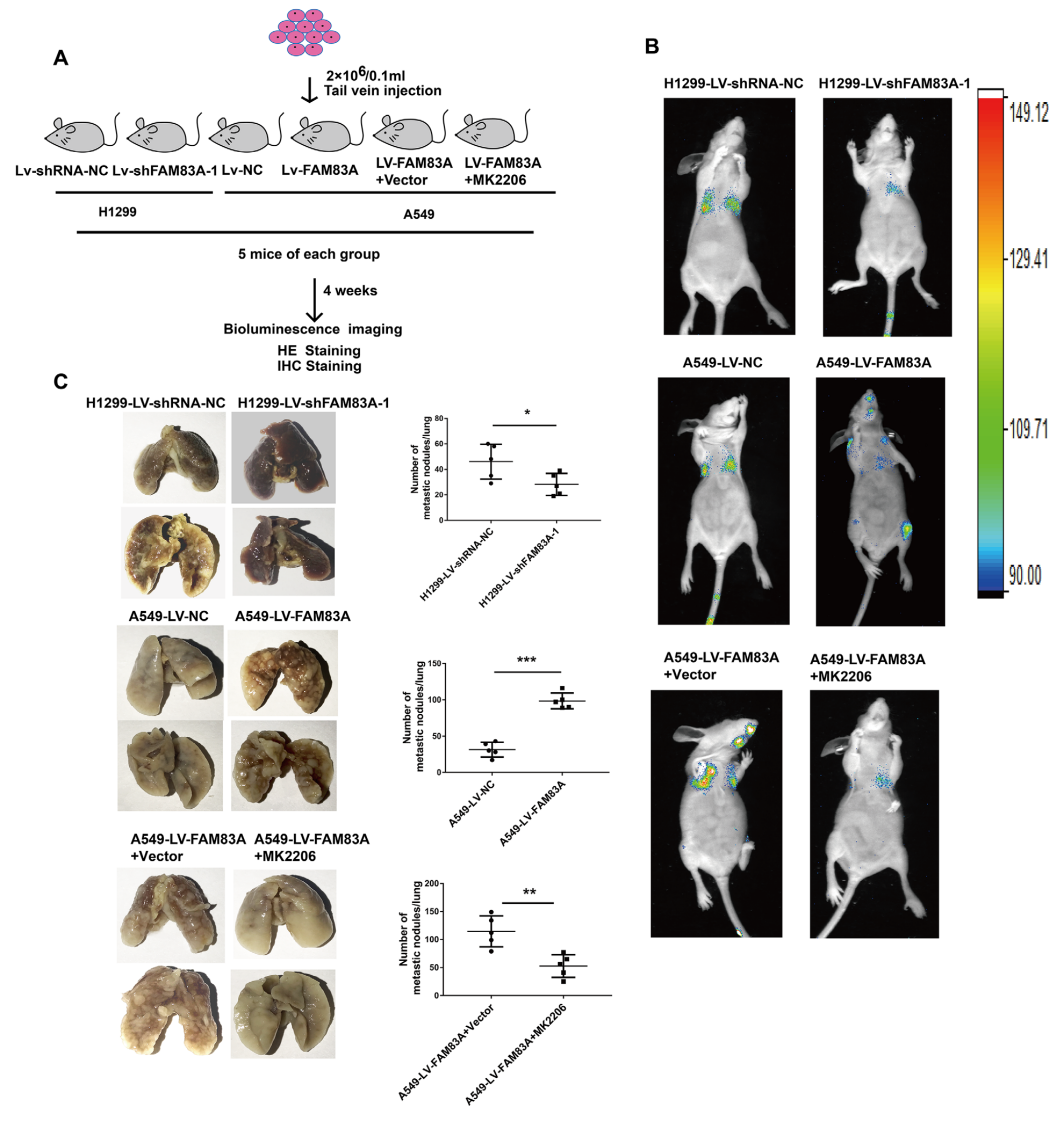
**Increased FAM83A expression promoted lung metastasis *in vivo,* and inhibition of AKT reduced the metastatic foci owing to FAM83A overexpression.** (**A**) Schematic diagram of the metastasis model in mice. (**B**) Stable H1299-LV-shFAM83A-1 or A549-LV-FAM83A cells (each also expressing luciferase) were transplanted into nude mice (tail vein injection). Two groups of nude mice overexpressing FAM83A were then treated with 30% Captisol diluents (Vector) or MK2206 at a dose of 50 mg/kg three times a week. Tumor formation in the lungs and distant metastasis were monitored by bioluminescence imaging. (**C**) Representative images and summary of the number of lung metastatic nodules. Error bars: mean ± SD (n=3). *p<0.05, **p<0.01, and ***p<0.001 were considered to indicate a statistically significant difference.

**Figure 9:**
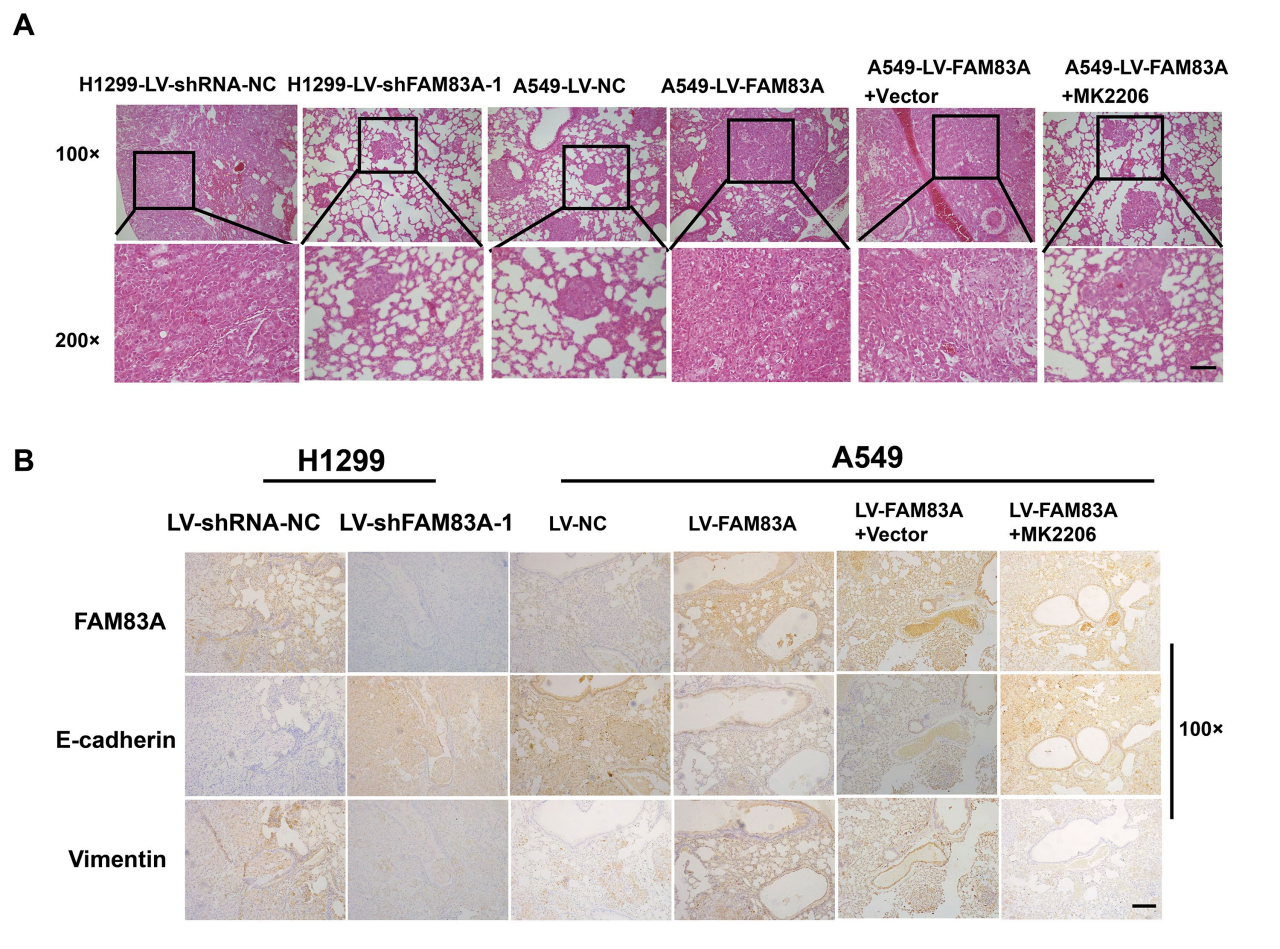
**Increased FAM83A expression promoted lung metastasis and EMT *in vivo,* and AKT inhibitor reduced EMT owing to FAM83A overexpression.** (**A**) HE staining of lung tissues in several groups (A549 and H1299 cells after manipulation of FAM83A expression with or without MK2206) of tumor-bearing mice. (**B**) Immunohistochemical staining for FAM83A, E-cadherin and Vimentin was performed using serial sections of mouse lung tissues. Scale bar, 100 μm.

To directly verify the role of FAM83A in metastasis *in vivo*, we conducted a separate analysis in nude mice by injecting stable FAM83A-knocked down H1299 or overexpressing A549 cells with luciferase expression in the tail vein (Figure 8A). The mice injected with stable FAM83A-depleted cells had markedly fewer metastasis (shFAM83A group, n=1/5) than did mice injected with stable mock-depleted cells (n=3/5). In contrast, the mice injected with stable FAM83A-overexpressing cell lines exhibited an increase in distant metastasis (n=5/5) involving the lungs, mandible and thighbone compared with mice injected with stable mock-transfected cells (n=3/5) (Figure 8B). Analysis of the number of metastatic lung nodes confirmed the enhanced colonization ability conferred by FAM83A (Figures 8C, 9A). The number of lung metastatic nodules was higher in A549-LV-FAM83A mice than in A549-LV-NC mice. In contrast, H1299-LV-shFAM83A mice showed fewer lung metastatic nodules than did H1299-LV-shRNA-NC mice (Figures 8C, 9A). Afterwards, mice with stable FAM83A-overexpressing were treated with an AKT inhibitor MK2206 (solubilized in 30% Captisol) or 30% Captisol diluents as vector control. We found MK2206 suppressed tumor metastatic foci compared with vector control (Figures 8B–8C, 9A), which indicated that the FAM83A/AKT pathway may be an effective therapeutic target in lung cancer metastasis. To further confirm that FAM83A promotes EMT *in vivo*, immunohistochemical staining (IHC) for FAM83A, E-cadherin and Vimentin was performed using serial sections of mouse lung tissues. As shown in the Figure 9B, we verified that E-cadherin (epithelium marker) was decreased and Vimentin (mesenchyme marker) was increased in mice with stable FAM83A-overexpressing. Inhibition of either AKT or FAM83A could impair the promoting effect of FAM83A overexpression in EMT. The promotion effect of FAM83A on lung cancer metastasis and EMT was further confirmed *in vivo*.

## DISCUSSION

Recent discoveries have revealed that the elevated expression of FAM83A occurs in a substantial fraction of cancers [23–28]. Indeed, observations have confirmed that FAM83A can promote anchorage-independent growth in mammary epithelial cells [24]. However, the specific oncogenic abilities and the molecular mechanism of FAM83A in EMT and metastasis of NSCLC remain largely unknown. In this study, we present evidence for the first time that overexpression of FAM83A occurs widely in NSCLC tissues and positively correlates with metastatic clinicopathological characteristics and a worse prognosis. Our findings are consistent with those reported by Liu et al, who demonstrated that FAM83A mRNA was overexpressed in the circulating tumor cells (CTCs) of lung adenocarcinoma patients [30]. Moreover, we found that forced FAM83A expression in NSCLC cells conferred enhanced invasive ability *in vitro*, resulting in significantly increased metastatic foci *in vivo*.

Up to now, the present study is the first to highlight the role of FAM83A in promoting EMT and metastasis in NSCLC. Prior studies have indicated that initiation of EMT in tumor cells is a major cause of lung cancer metastasis [33–36]. Here, we found FAM83A promoted the EMT process both *in vitro* and *in vivo*. Moreover, depletion of FAM83A decreased Snail expression but did not affect Twist expression. This suggested that FAM83A enhanced EMT by inducing Snail but not Twist. Meanwhile, we proved that knocking down Snail could reduce the increase of migration and invasion caused by FAM83A overexpression, which further confirmed that FAM83A promoted EMT through Snail. Afterwards, we explored the signaling pathway that could manipulate the expression of Snail. PI3K/AKT pathway is a common signaling used to manipulate the EMT process [18]. Prior studies have indicated that the constitutive activation of PI3K/AKT signaling cascades is closely correlated with the upregulation of Snail and the metastasis of diverse tumor cells [37–41]. Moreover, Lee et al. [29] reported that FAM83A can trigger the PI3K/AKT pathway to facilitate the progression of breast cancer. In the present study, we demonstrated the FAM83A/PI3K/AKT pathway promoted Snail-induced EMT and metastasis. These results were further confirmed by pharmacologically blocking the PI3K/AKT signaling pathway by using the AKT inhibitor MK2206. Besides, overexpression of FAM83A following the inhibition of AKT can restore the metastasis ability of cells, as well as the expression of p-AKT (Supplementary Figure 2A–2D). In conclusion, our study demonstrated FAM83A could promote metastasis via the PI3K/AKT/Snail pathway.

We believe there may be other mechanisms to promote metastasis by FAM83A that need to be further explored despite of PI3K/AKT pathway. Studies have shown that FAM83A can also interact with MAPK signaling pathway [24, 29]. Shi et al. proposed FAM83A-S1 could promote the development of lung cancer through the FAM83A/ERK pathway [31]. We also confirmed that MAPK signal could promote metastasis in FAM83A overexpressing lung cancer cells by applying ERK inhibitor AZD8330 (Supplementary Figure 3A–3F). It is possible that MAPK pathway also contributes to the metastasis of lung cancer with FAM83A expression. However, we proposed a new FAM83A pathway (PI3K/AKT pathway) promoting lung cancer metastasis despite of the MAPK pathway. Moreover, our data indicated that inhibition of AKT or ERK only partially suppressed the metastasis. We believe that there may some other mechanisms to be further explored in FAM83A promoting metastasis. Fulcher LJ et al. found FAM83A could interact with the casein kinase 1 family ɛ (CK1 ɛ), restricting the function of CK1 enzymes in cells [21]. Xu et al. revealed CK1ɛ could phosphorylate the β-TrCP recognition region of Snail in collaboration with GSK3β. depletion of CK1ɛ can impair the phosphorylation and degradation of Snail and promotes cell migration [42], suggesting a feasible role of CK1ɛ in the EMT process. Thus, we wonder that whether could FAM83A limit the function of CK1 ɛ in cells and make it fail to degrade Snail, thereby promote the occurrence of EMT.

Except for the effect on metastasis, we found that the proliferation ability of cells decreased after inhibiting FAM83A (Supplementary Figure 4). This is consistent with the results of Shi et al, which demonstrated that long noncoding antisense RNA FAM83A-AS1 promoted lung cancer cell proliferation by increasing FAM83A in lung cancer cell line A549 [31]. The effect of FAM83A on proliferation may be due to its location in 13q24, a region containing MYC, which is closely related to proliferation. They may have similar functions due to locating in the same area.

Overall, our present study identified FAM83A as a new pro-metastatic factor in NSCLC. We suggested FAM83A in cancer as a key metastasis promoter by facilitating PI3K/AKT/Snail-mediated EMT and metastasis. FAM83A expression is barely detected in normal peripheral lung tissues, its high expression in lung cancer tissues makes it tumor-specific. Our study offers an expanded therapeutic window compared to direct PI3K/AKT inhibition, since they are required for normal cellular homeostasis. These findings give new insight into a potential oncogenic role for FAM83A in NSCLC progression and may provide a novel therapeutic target in NSCLC treatment.

## MATERIALS AND METHODS

### Ethics statement

Investigation has been conducted in accordance with the ethical standards and according to the Declaration of Helsinki and according to national and international guidelines and has been approved by the Institutional Review Board of The Harbin Medical University.

### Data source and bioinformatics analysis

The cluster analysis data used in this study were obtained from The Cancer Genome Atlas database (https://cancergenome.nih.gov). The GEPIA (http://gepia.cancer-pku.cn/index.html), a newly developed interactive web server used to analyze samples from the TCGA and GTEx projects, was utilized with a standard processing pipeline [43]. By GEPIA, we examined the expression of FAM83A in NSCLC cancer and normal tissues. A boxplot was constructed to visualize the relationship. Then, we analyzed the overall survival and disease-free survival of patients with tumors expressing different levels of FAM83A in lung cancer. Pearson’s test was used to discover the correlation between FAM83A and Snail in NSCLC tissues.

### Clinical NSCLC samples and immunohistochemistry

All experimental protocols were approved by the Institutional Review Board of The Harbin Medical University. A total of 101 paraffin-embedded NSCLC samples that were histopathologically and clinically diagnosed were obtained post-operatively from the Harbin Medical University Cancer Hospital between 2008 and 2012, and immunohistochemical staining was then performed using a FAM83A antibody (1:100; Sigma, Saint Louis, MO, USA). The data were analyzed anonymously. Briefly, sections were deparaffinized with xylene followed by ethanol and then washed with distilled water. For antigen retrieval, sections were submerged into EDTA antigenic retrieval buffer, microwaved, and then blocked with 3% hydrogen peroxide for 10 min at room temperature, followed by incubation with Serum-Free Protein Block (DAKO, Glostrup, Denmark) for 60 min to block nonspecific binding. The slides were incubated overnight at 4°C with the indicated primary antibodies (the IgG antibody was used as a negative control). After washing, the tissue sections were treated with a peroxidase-labeled secondary antibody (Zymed, San Francisco, CA, USA). The sections were then rinsed in PBS, stained with DAB (liquid DAB + substrate, DAKO, Glostrup, Denmark) and counterstained with hematoxylin.

The stained slides were examined by two independent observers in a blinded fashion. The scores were determined by combining the intensity of staining and the proportion of positively stained cells. The intensity score (IS) was based on FAM83A cytoplasm staining as follows: 0, no staining; 1, weak and incomplete staining; 2, moderate to complete staining; or 3, strong and complete, homogenous staining. Cell proportions were scored as follows: 0, no positive cells; 1, <10% positive cells; 2, 10–35% positive cells; 3, 35–75% positive cells; and 4, >75% positive cells. We then calculated the staining index (SI) by multiplying the IS by cell proportions. SI from 0 to 3 was defined as low expression, SI greater than or equal to 4 defined as a high expression.

### Cell culture

A panel of human lung cancer cell lines, PC14, H661, A549, H827, PC9, H1915, H2170, H460 and H1299, was used for *in vitro* validation and functional analysis. These cells were purchased from the ATCC (Manassas, VA) and cultured in RPMI medium (HyClone, Logan, UT) Aldrich, St. Louis, MO) in an atmosphere at 37°C in a humidified 5% CO_2_ incubator. The cells with gene transfection were treated with or without 1μM MK2206 (AKT inhibitor) (Chemietek, Indianapolis, IN) for 24h [44] / 50nM AZD8330 (ERK inhibitor) (Selleckchem,Texas, USA) for 18h [45], which were dissolved in dimethylsulfoxide (10 mM stock solution) and stored at 20°C. The drug was used at the indicated final concentrations in culture medium.

### Lentivirus transduction and generation of stable cell lines

The human FAM83A lentivirus (LV-FAM83A), the negative control (LV-NC), the shRNA lentivirus targeting FAM83A (LV-shFAM83A-1/2), and the scrambled shRNA lentivirus (LV-shRNA-NC) were purchased from JIKAI company (Shanghai, China). The target sequences of FAM83A were 5′- GCCGCCTTAGCAGCAGCAGT-3′ for LV-shFAM83A-1, 5′-CCGCCTTAGCAGCAGCAGT -3′ for LV-shFAM83A-2 and 5′- CAACAAGATGAAGAGCACCAA -3′ for LV-shRNA-NC. Stable cells were selected with 4mg/mL puromycin (Beyotime, Nanjing, China) after infection. Positive clones were then selected and amplified for further analyses.

### siRNA transfection

To inhibit the expression of Snail, cells with FAM83A overexpression were transfected with Snail-specific siRNAs (siSnail-1, 5′- AGTTTATTGATATTCA ATA -3′; siSnail-2, 5′- TGGTTAATTTATATACTAA -3′ or non-specific siRNA (Vector), 5′-TTCTCCGAACGTGTCACGTAA-3′ (10 μmol/L) synthesized by JIKAI company (Shanghai, China). Cells were transfected with 50 nM siRNA using Lipofectamine 2000 (Invitrogen, Carlsbad, CA) according to the manufacturer’s instruction. Experiments were performed 48 hours post-transfection. Non-specific siRNA was used as vector control.

### Real-Time RT-PCR analysis

Total RNA was isolated from cells using an RNeasy Mini Kit (Qiagen Ltd., Germany). Reverse transcription was carried out using the Prime Script RT Reagent Kit (Takara, Japan) according to the manufacturer’s protocol. qPCR was then performed using SYBR Green (ROX; Roche, Toronto, ON, Canada) in Stratagene MX3000P (Agilent Technologies, Santa Clara, CA, USA). Primers were synthesized by IDT, and calculations were performed using the values of the average cycle threshold (Ct) as the calibrator and calculated as 2–[(Ct of gene)–(Ct of GAPDH)]. Each assay was performed at least in biological and PCR triplicate. The primers used (FAM83A-F and FAM83A -R; GAPDH-F and GAPDH-R) as follow: FAM83A sense 5′-CTCGGACTGGAGATTTGTCC-3′; FAM83A anti-sense 5′-GGAACTCCTCGTCAAACAGC-3′; GAPDH sense 5′-CTCCTCCTGTTCGACAGTCAGC -3′; GAPDH anti-sense 5′- CCCAATACGACCAAATCCGTT -3′.

### Western blot analysis

Monolayer cells were washed with PBS and lysed in radioimmunoprecipitation assay (RIPA) buffer (Thermo Scientific, Carlsbad, California, USA) containing a protease inhibitor cocktail (Selleck, Huston, Texas, USA). Protein concentration was determined with a BCA protein assay (Thermo Scientific, Carlsbad, California, USA) and equal amounts of proteins was resolved by sodium dodecyl sulfate polyacrylamide (SDS-PAGE) gel and transferred to the PVDF membrane (Millipore, Billerica, MA). Total cell lysates (60 μg) were assayed by Western blot analysis using standard procedures. Western blot membranes were incubated overnight at 4°C with the following primary antibodies: anti-FAM83A (1:1000; Sigma, Saint Louis, MO, USA); anti-β-actin (1:10000; Abcam, Cambridge, USA); anti-PI3K(1:5000; Abcam, Cambridge, USA); anti-phosphorylated PI3K p85 subunit (p-Y458) (1:1000; Cell Signaling Technology, Danvers, MA, USA); anti-phosphorylated AKT (p-S473) (1:5000; Abcam, Cambridge, USA); anti-Akt (1:5000; Abcam, Cambridge, USA); anti-Vimentin(1:1000; Cell Signaling Technology, Danvers, MA, USA); anti-E-cadherin (1:1000; Cell Signaling Technology, Danvers, MA, USA); anti-Snail (1:1000 Cell Signaling Technology, Danvers, MA, USA); anti-Twist (1:500 Cell Signaling Technology, Danvers, MA, USA); anti-phosphorylated Erk1/2 (Thr202/Tyr204) (1:2000; Cell Signaling Technology, Danvers, MA, USA) and Erk1/2 (1:1000; Cell Signaling Technology, Danvers, MA, USA). Afterwards, HRP-conjugated secondary antibodies (DAKO, Glostrup, Denmark) were used for detection on an Odyssey machine (Roche Diagnostics, Hilden, Germany) with ECL detection reagent (Amersham Biosciences, Castle Hill, Australia). Protein levels in all experiments were normalized to those of β-actin.

### Wound-healing assays

For the wound-healing assays, cells were seeded into a 6-well plate at a density of 2 × 10^5^ cells per well and grown overnight until they reached 80~95% confluence as a monolayer. A wound was created by gently and slowly scraping the cell monolayer with a 10 μL pipette tip across the center of the triplicate well. After scratching, the well was replenished with fresh serum-free medium and the cells were treated with 1ug/ml Mitomycin (Biolab, Peking) for one hour in advance. Wound closure was observed within 24h, the percentage of closure was calculated. Images were obtained at various time points after wounding.

### Cell migration and matrigel invasion assays

Cells were trypsinized, resuspended in medium without serum and plated at a density of 2*10^4^ cells/well in a Transwell insert (8mmpore size, BD Biosciences, USA) for the migration assay, and 4*10^4^ cells/well were plated in a Matrigel invasion chamber (8 mm pore size, BD Biosciences, USA) for the invasion assay. The chemoattractant in the lower chamber was 10% fetal bovine serum. After a 6-h incubation for the migration assay or a 22-h incubation for the invasion assay, the uninvaded cells were removed by mechanical scraping, while invaded cells were fixed with 4% PFA for 1 h and then stained with 0.1% crystal violet. Invaded cells were visualized under a inverted microscope (Leica DMI 4000 B, Wetzlar, Germany). Cell numbers were quantified manually.

### Immunofluorescence staining

Transfected NSCLC cells were seeded onto cover slips and grown to 50-70% confluency. Cells were then washed with PBS, fixed with 4% paraformaldehyde, permeabilized with or without 0.3% Triton-X, and blocked with 5% normal goat serum at 37°C for 30 min. The antibodies against E-cadherin (1:200 Cell Signaling Technology, Danvers, MA, USA) or Vimentin (1:100; Cell Signaling Technology, Danvers, MA, USA) were added and incubated at 4°C overnight. After incubation with rabbit anti-E-cadherin and mouse anti-Vimentin antibodies, followed by incubation with Alexa Fluor™488-conjugated goat anti-mouse IgG (1:1000; Life Technologies, Carlsbad, California, USA) and Alexa Fluor™ 594-conjugated goat anti-rabbit IgG (1:1000, Life Technologies, Carlsbad, California, USA) at 37°C for 1 h, the cells were washed three times with PBS and stained with DAPI (Life Technologies, Carlsbad, California, USA). Images were acquired by OLYMPUS FV1000.

### Cell proliferation assay

The cell viability were detected using CCK-8 assay as following: Cells were cultured at a density of 1000 cells/well in 96-well plates with advanced RPMI 1640 medium supplemented with 10% FBS at 5% CO2,37°C. Every 24 h, CCK-8 (Sigma Chemical Co) was added at a final concentration of 0.5 mg/mL and absorbance was measured at 450 nm. All assays were performed in triplicate and repeated at least three times.

### *In vivo* metastasis assay

All animal experiments were approved by the animal care Committee of Harbin Medical University. BALB/c (nu/nu) female nude mice (5 weeks of age, 18–20 g), purchased from Slac Laboratory Animal of Chinese Academy of Science (Shanghai, China), were randomly divided into 4 groups (n=5 per group). Mice were subjected to1 week of adaptation (housed with sterilized cages under a 12-h light/dark cycle at 18–22°C and 50–60% relative humidity). Food and drinking water were provided adlibitum. One hundred microliters of PBS containing 2.0 × 10^6^ A549 cells with or without FAM83A overexpression and H1299 cells with or without FAM83A knockdown (shFAM83A-1) were injected into the tail vain of each mouse. Metastatic lesions were monitored every week using bioluminescence imaging (BLI). Briefly, the mice were anesthetized and injected intraperitoneally with 150 μg of D-luciferin (Yeasen, Shanghai, China) per gram of weight. After 5 min, the bioluminescence was imaged using an IVIS imaging system (Caliper Life Sciences, Alameda, CA, USA) and analyzed using Living Image Software 4.3.1. Four weeks later, the injected nude mice were sacrificed, and the tumor tissues were excised and fixed in 4% paraformaldehyde solution for further HE and immunohistochemical staining analysis. All experimental procedures were conducted in accordance with the Guide for the Care and Use of Laboratory Animals and conformed to our institutional ethical guidelines.

### Determination of the therapeutic effects of the AKT inhibitor

BALB/c (nu/nu) female nude mice (5 weeks of age, 18–20 g), purchased from Slac Laboratory Animal of Chinese Academy of Science (Shanghai, China), were randomly divided into 2 groups (n=5 per group). One hundred microliters of PBS containing 2.0 × 10^6^ A549 cells with FAM83A overexpression were injected into the tail vain of each mouse. Afterwards, the AKT inhibitor MK2206 was formulated in 30% Captisol (Cydex, La Jolla, CA, USA) and administered by oral at gavage thrice a week in the first group. The control group was treated with 30% Captisol by oral gavage thrice a week. Based on doses previously used in a clinical trial, we choose 50 mg/kg 3 times a week [46]. Tumor metastasis, HE and immunohistochemical staining were analyzed after 4 weeks.

### Statistical analysis

All values are reported as the mean and standard deviation (SD) of at least three independent experiments, which were performed in biological triplicate and repeated twice. Survival curves were performed using the Kaplan-Meier method. Univariate and Multivariate statistical analysis was performed with a Cox regression model. Data were analyzed using Student’s *t*-test; a *P* value < 0.05 was required for statistical significance. All statistical analyses were performed with SPSS 19.0 software (SPSS, Inc., Chicago, IL).

## Supplementary Material

Supplementary Figures
