# Supplementary material for: FAM83A signaling induces epithelial-mesenchymal transition by the PI3K/AKT/Snail pathway in NSCLC
Source: Aging (Albany NY). 2019 Aug 24;11(16):6069–88. doi: 10.18632/aging.102163 (PMC6738414; doi:10.18632/aging.102163)
Supplement: Supplementary Figures [file aging-11-102163-s001.pdf]

SUPPLEMENTARY FIGURES

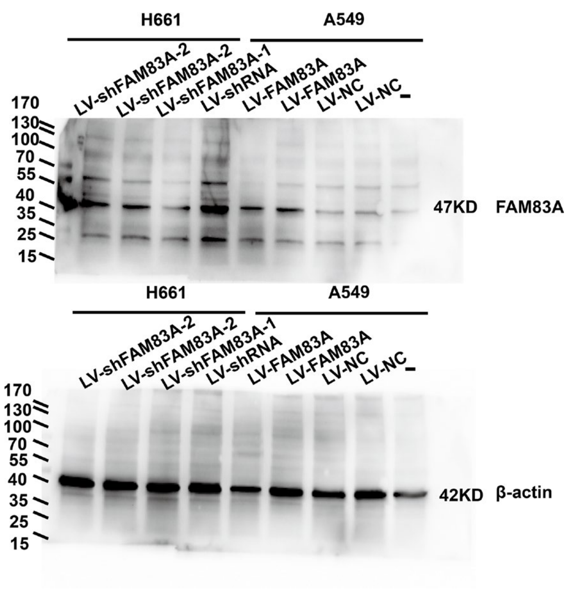

Supplementary Figure 1. A full membrane with the FAM83A knockdown and overexpression cell lines by Western Blot.

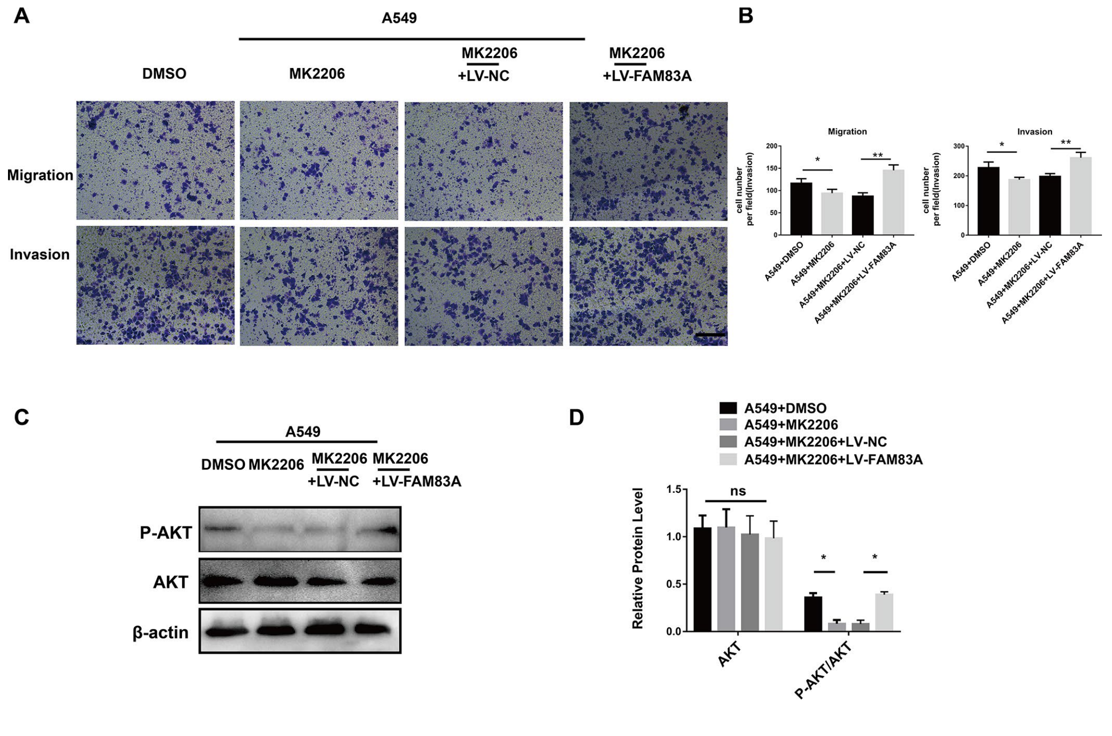

Supplementary Figure 2. Overexpression of FAM83A after inhibition of AKT restored the migration and invasion ability, as well as the expression of p-AKT in cell. (**A**, **B**) Firstly, A549 cells were treated by MK2206 and then FAM83A was overexpressed. These cells along with A549 cells (with or without MK2206) were subjected to a Transwell assay. (**C**, **D**) Protein levels of AKT and P-AKT (p-S473) were detected by Western blot analysis. Scale bar, 200 μm. Error bars: mean ± SD (n=3). NS, no significant, *p<0.05 and **p<0.01 were considered to indicate a statistically significant difference.

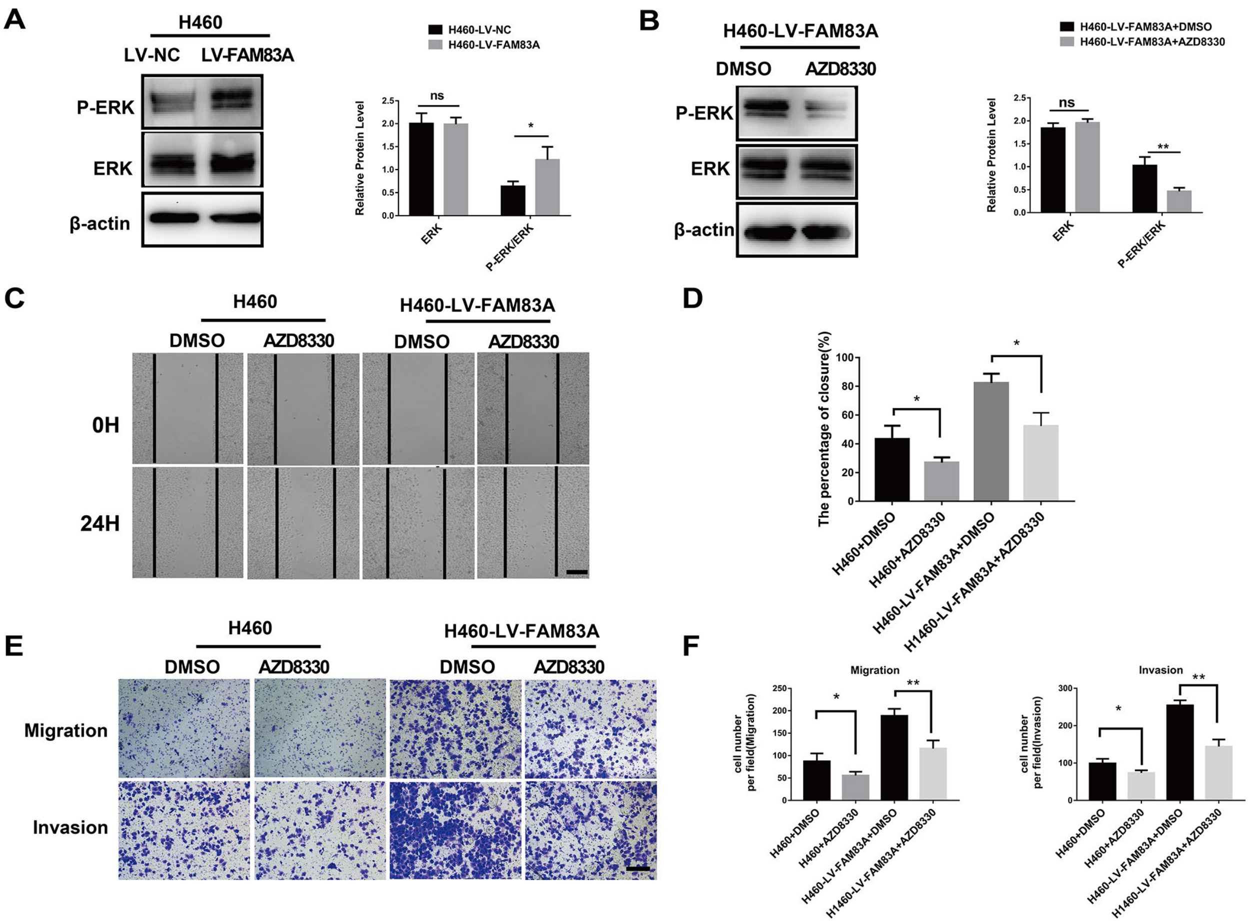

Supplementary Figure 3. The role of MAPK signal pathway in promoting metastasis in FAM83A overexpression lung cancer cells. (**A**) Protein levels of ERK1/2 and phosphorylated ERK1/2 (p-T202/Y204) were detected by Western blot in stable H460-LV-NC and H460-LV-FAM83A cells. β-actin was used as a loading control for ERK. (**B**) Protein levels of ERK1/2 and phosphorylated ERK1/2 (p-T202/Y204) were detected by Western blot in stable H460-LV-FAM83A cells (with or without ERK inhibitor AZD8330). (**C**–**F**) H460 cells and H460-LV-FAM83A cells (with or without AZD8330) were subjected to a wound-healing assay (**C**, **D**) and a Transwell assay (**E**, **F**). Scale bar, 200 µm. Error bars: mean ± SD (n=3). NS, no significant, *p<0.05 and **p<0.01 were considered to indicate a statistically significant difference.

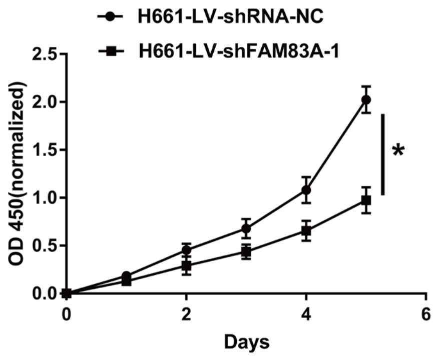

Supplementary Figure 4. FAM83A promoted cell proliferation in lung cancer cell. The cell viability was inhibited in FAM83A-knockdown cell compared with control. Error bars: mean ± SD (n=3). *p<0.05 was considered to indicate a statistically significant difference.
